# Assessment of the Behavioral and Neurochemical Characteristics in a Mice Model of the Premotor Stage of Parkinson’s Disease Induced by Chronic Administration of a Low Dose of MPTP

**DOI:** 10.3390/ijms26188856

**Published:** 2025-09-11

**Authors:** Yulia A. Timoshina, Anastasia K. Pavlova, Dmitry N. Voronkov, Denis A. Abaimov, Alexander V. Latanov, Tatiana N. Fedorova

**Affiliations:** 1 School of Biology, Lomonosov Moscow State University, 119991 Moscow, Russia; pav_nastasya@mail.ru (A.K.P.); latanov.msu@gmail.com (A.V.L.); 2 Russian Center of Neurology and Neurosciences, 125367 Moscow, Russia; voronkovdm@gmail.com (D.N.V.); abaidenis@yandex.ru (D.A.A.); tnf51@bk.ru (T.N.F.); 3 Research Institute for Brain Development and Peak Performance, Peoples’ Friendship University of Russia (RUDN), 117198 Moscow, Russia

**Keywords:** parkinsonism, early stage, MPTP, spatial learning, Na,K-ATPase, cytochrome oxidase, antioxidant system

## Abstract

Parkinson’s disease is the second most common neurodegenerative movement disorder caused by the death of dopaminergic neurons in the Substantia nigra. The motor symptoms of Parkinson’s disease only become apparent in the late stages, whereas non-motor impairments often manifest earlier. Therefore, devising adequate experimental models to study the pathogenesis of Parkinson’s disease is of fundamental scientific importance. In this study, we aimed to evaluate the behavioral and neurochemical characteristics in a model of the premotor stage of parkinsonism in mice induced by chronic administration of a low dose of methyl-4-phenyl-1,2,3,6-tetrahydropyridine MPTP. Administering 3 mg/kg of the toxin for 35 days does not cause motor deficits, except in fine motor skills, and results in impaired spatial learning. In addition, this stage is characterized by the depletion of striatum and prefrontal cortex dopamine, decreased tyrosine hydroxylase in striatum and Substantia nigra, increased cytochrome oxidase and superoxide dismutase expression, and microglia activation. Concluding, the presented model made it possible to identify a complex of physiological and neurochemical disorders characteristic of the early stage of Parkinsonism.

## 1. Introduction

Parkinson’s disease is a progressive neurodegenerative disorder characterized by the loss of dopaminergic neurons in the substantia nigra pars compacta (SNpc), and subsequent dopamine depletion in the striatum, ultimately leading to motor deficits. The diagnostic features of Parkinson’s disease (PD) are divided into motor and non-motor dysfunctions. The main motor symptoms of Parkinson’s disease include rest tremor, bradykinesia, rigidity, and postural instability. Non-motor symptoms include dysautonomia, cognitive and neurobehavioural impairments, sleep disorders and sensory deficits [1].

The main symptoms of the disease become clinically diagnosable when the neuronal death in the SNpc reaches 50–60% and is accompanied by a 70% decrease in dopamine levels. At the same time, the absence of any pronounced symptoms at the premotor stage of the disease suggests some powerful compensatory mechanisms that can mitigate this significant depletion of the dopaminergic system [2,3]. Moreover, striatal denervation and neuronal death in the SNpc are known to occur exponentially pattern and still persist for around 5 years following the diagnosis [4].

Modern experimental models of Parkinson’s disease are primarily neurotoxin-based. They administer compounds such as 6-hydroxydopamine (6-OH-DA), 1-methyl-4-phenyl-1,2,3,6-tetrahydropyridine (MPTP), or rotenone induces oxidative stress (OS) and cell death in the dopaminergic neuronal population, which mimics sporadic PD [5]. The advantage of models based on the introduction of MPTP is that they reproduce neurodegeneration quite accurately; a decrease in striatal dopamine occurs after the death of dopaminergic neurons in the substantia nigra and degeneration of their terminals, with neuron loss concentrated in the ventral, lateral, and posterior regions of the SNpc, while the anterior and medial regions remain functional [6]. Upon administration into the animal body, MPTP is oxidized to form the toxic metabolite 1-methyl-4-phenyl-pyridinium ion (MPP+), which is absorbed by dopaminergic neurons through the dopamine transporter (DAT). MPP+ inhibits the function of the complex I of the mitochondrial electron transport chain (ETC), leading to a rapid decrease in ATP in the striatum and SNpc, followed by apoptosis and necrosis of dopaminergic neurons and a decrease in the dopamine level [7]. Unlike a neurotoxin 6-OH-DA, MPTP crossed blood–brain barrier after systemic administration and exhibited the notable degeneration of nigrostriatal dopaminergic neurons in a PD model and has significantly lower overall toxicity compared to paraquat and rotenone [8]. In turn, the toxins paraquat and rotenone, which are also used to model Parkinson’s disease, have a general toxic effect, and often the motor disorders that occur when paraquat and rotenone are administered do not correlate with the depletion of striatal dopamine and the death of neurons in the SNpc [9,10]. These facts allow researchers to develop models for the long-term development of neurodegeneration with chronic MPTP administration, while models using 6-OH-DA and rotenone are effective in acute modeling of parkinsonian-like condition. However, with certain protocols for administering 6-OH-DA and rotenone to the medial forebrain bundle, medial ventral tegmental area, or SNpc, it is possible to achieve the presymptomatic stage of Parkinson’s disease with the development of characteristic non-motor symptoms, such as the formation of depression-like behaviour, anhedonia, and apathy [11,12]. Nevertheless, the use of a protocol for prolonged administration of low doses of the neurotoxin MPTP allows the progression of dopamine neuron degeneration to be modelled.

Models of long-term neurodegeneration allow us to study the possible mechanisms of the onset and course of Parkinson’s disease at different stages of development. The growing proportion of neurodegenerative diseases in the population urgently poses the task of finding their molecular mechanisms, as well as effective means of prevention and treatment. Recently, researchers have been considering Na,K-ATPase as one of the potential agents responsible for the onset of PD. A large amount of evidence points to the association of Na,K-ATPase dysfunction with the development of Parkinson’s disease. For example, mutations in the ATP1A3 gene cause rapid onset dystonia-parkinsonism (RDP) and alternating hemiplegia childhood (AHC) [13]. Neurotoxic α-synuclein aggregates, which are a hallmark of Parkinson’s disease, bind to the Na,K-ATPase neuronal α3 subunit, disrupting its function [14]. Oxidative stress, which can be caused by toxic dopamine metabolites, also causes neuronal Na,K-ATPase dysfunction [15]. Thus, there is reason to study the role of Na,K-ATPase dysfunction in pathophysiological processes in the central nervous system (CNS). It has been shown that in the late stages of toxin-induced parkinsonism, there is a significant decrease in Na,K-ATPase activity [16]. In this regard, the relevant question is can Na,K-ATPase dysfunction lie at the beginning of the pathological process, or does a decrease in its pumping function occur a second time?

The progression of neurodegeneration and symptom development depends directly on the toxin administration protocol. In early studies, MPTP was administered via repeated injections of 20 mg/kg at 2-h intervals over the course of 2 days or 10 mg/kg for 1 day with an interval of 1 h. This protocol induced the onset of the key parkinsonism symptoms (tremor, rigidity, bradykinesia) and a 50–60% loss of SN dopaminergic neurons as well as a 50% mortality rate in experimental animals [17,18,19]. When the toxin is administered sub-acutely at a dose of 30 mg/kg (once a day over the course of 5 days), fewer neurons are degraded as compared to the acute administration, with insignificant animal mortality (5% on average). However, in most cases animals do not develop the characteristic motor symptoms [20,21,22]. Moreover, the presented above models exhibit a rapid progression of pathology and behavioral disorders, making them unsuitable for a detailed temporal assessment of disease dynamics.

Therefore, models of chronic administration of toxins, primarily MPTP, are becoming increasingly relevant. To date, several methods of modeling the long-term parkinsonism utilizing this compound have been described. First, a daily administration of MPTP at a dose of 4 mg/kg for 20–28 days [17,22,23]. Second, fractional administration of MPTP at a dose of 20–30 mg/kg several times a week (usually 2.5–3 times) over the course of 1 to 3 months [24], and fractional administration of MPTP at a dose of 25 mg/kg combined with probenecid, which reduces renal excretion of the toxin, thereby prolonging its affective time at a dose of 250 mg/kg (2.5–3 times a week) over the course of 5 weeks [25]. Another study involved MPTP infusion via osmotic pumps at a dose of 5 mg/kg or 30 mg/kg over the course of 28 days [26]. As MPTP exhibits a relatively rapid pharmacotoxicity and becomes undetectable in the body after 8 h [27], the proposed chronic administration models entail repeated acute exposure of dopaminergic neurons, leading to extensive OS.

In view of the above, a promising approach is to use low doses of the toxin to create a mild precision impact, activate the internal protective mechanisms and trace the dynamics of the neurodegenerative process. Previously, the dynamics of dopaminergic neuron death in the SNpc, as well as the progression of the antioxidant system dysfunction of the brain in the model of chronic subcutaneous MPTP administration at a dose of 4 mg/kg for 23 days, were described in detail. The authors have demonstrated a reduced SNpc neuron number by day 3 as well as the two-phase nature of a decrease in the striatal dopamine, which was observed on days 1 and 23 of toxin administration. These events were accompanied by a gradual decline in the antioxidant system function starting from day 3 [22]. At the same time, assessing the behavioral and neurochemical features at the premotor stage of the disease upon chronic administration of lower doses of the toxin is of particular interest.

In this regard, developing a model of the early pre-symptomatic parkinsonism, characterized by the presence of non-motor manifestations with a slowly progressing neurodegeneration that closely resembles the actual course of the disease is a task of utmost importance. This chronic MPTP-model should be useful for the evaluation compensatory mechanisms of the dynamic physiopathological changes which occur at different stages of PD.

This research aimed to evaluate behavioral and neurochemical characteristics in the model of pre-motor stage of Parkinson-like syndrome in mice, induced by the chronic administration of low doses of the MPTP neurotoxin.

## 2. Results

### 2.1. Evaluation of Motor and Non-Motor Deficits

**Figure 1:**
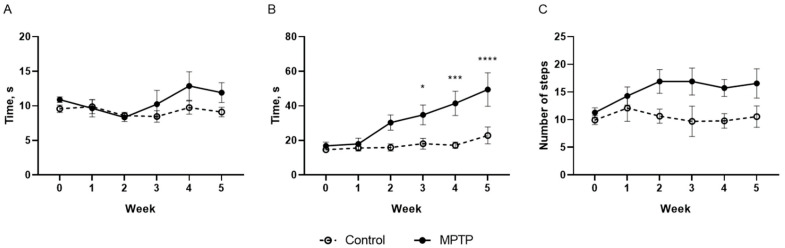
Time required to pass the beam walking test (**A**). Time required to pass the inclined grid walking test (**B**). The number of mistakes in the inclined grid walking test (**C**). N = 12 in the control group and N = 11 in the MPTP group. The data are presented as mean ± SEM. The significance of the differences between the groups was compared using the two-way ANOVA analysis with Sidak correction for multiple comparisons, *-*p* < 0.05, ***-*p* < 0.001, ****-*p* < 0.0001—as compared to the control group.

The presence of motor symptoms was assessed in a set of tests that allowed for estimating coordination, bradykinesia, and fine motor skills in the animals. The animals from both groups showed the same time during the Beam walking test (Figure 1A), indicating that chronic administration of 3 mg/kg of MPTP did not affect animals’ balance and coordination. Starting from the third week of the experiment, the animals that received MPTP showed worse performance in the inclined grid walking test (Figure 1B). In the third week, the experimental animals passed the test two times slower than control animals (*p* = 0.0293, F(1, 100) = 22.6), 2.4 times (*p* = 0.0010, F(1, 100) = 22.6) and 2.2 times slower (*p* < 0.0001, F(1, 100) = 22.6) in the 4th and 5th weeks, respectively. This parameter in the control group was the same. At the same time, there was no statistically significant difference in the number of mistakes made during the inclined grid walking test between the groups (Figure 1C). Thus, no motor symptoms were observed after 35 injections of the toxin, based on the test results. However, the grid walking test revealed a decrease in the animals’ speed, indicating the onset of bradykinesia. Taken together, the obtained results serve as an evidence that this model accurately describes the early stage of parkinsonism.

**Figure 2:**
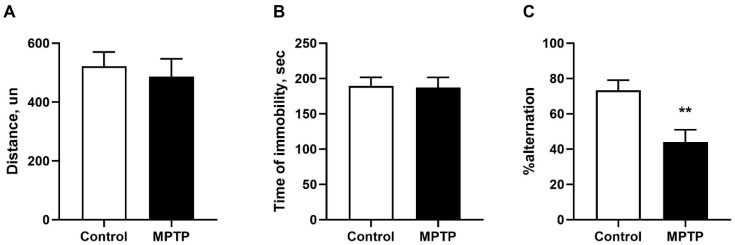
Total distance traveled in the open field test (**A**). The total duration of immobility in the tail suspension test (**B**). Evaluation of spatial working memory impairment in the Y-maze test (**C**). N = 12 in the control group, N = 11 in the MPTP group for open field test, N = 7 in the control group, and N = 6 in the MPTP group for tail-suspension test and Y-maze test. The data are presented as mean ± SEM. Groups were compared by *t*-test, **-*p* < 0.01 as compared to the control group.

The locomotor activity (distance traveled) of MPTP animals in the open field test was not differ from the control values (Figure 2A). No differences in mouse exploratory or rearing behavior were discovered in this test. The analysis of behavior in the tail-suspension test also revealed no differences between the experimental groups (Figure 2B). Spatial memory impairment after 35 injections of MPTP was evaluated using the Y-maze test by the number of maze arm visits by the animals. The animals from the control group made correct choices of maze arms in 73% of cases, while the animals receiving MPTP chose correct arms only in 43% (*p* = 0.009, F = 1.512) and showed a higher number of repeated entries into the arms. These results demonstrate that MPTP disrupts spatial working memory (Figure 2C).

### 2.2. The Content of Dopamine and Its Metabolites in Mice’s Striatum and Prefrontal Cortex

**Figure 3:**
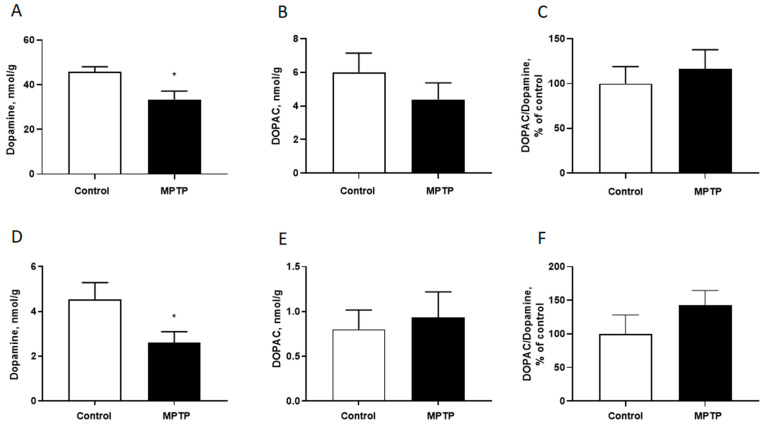
The content of dopamine (**A**), 3,4-Dihydroxyphenylacetic acid (DOPAC) (**B**), the DOPAC/Dopamine ration (**C**) in the striatum and in the cortex (**D**–**F**, respectively). N = 12 in the control group, N = 11 in the MPTP group. The data are presented as mean ± SEM. Groups were compared by *t*-test, *-*p* < 0.05 as compared to the control group.

By the end of the experiment, the striatal dopamine content in the experimental group animals decreased by 34% relative to the control group (*p* = 0.0262, F = 3.960) (Figure 3A). There were no statistically significant changes in the DOPAC content (*p* = 0.3023, F = 1.076) (Figure 3B) and the DOPAC/dopamine ratio (*p* = 0.3432, F = 2.927), which reflect the metabolic processes of dopamine turnover (Figure 3C). Administering MPTP for 35 days led to a 52% decrease in the dopamine content in the PFC compared to the control group (*p* = 0.0380, F = 5.588) (Figure 3D). However, there were no statistically significant changes in the content of DOPAC (*p* = 0.7095, F = 1.566) (Figure 3E) and the ratio of DOPAC/Dopamine (*p* = 0.4844, F = 1.206) (Figure 3F) by the end of the experiment. There were no differences in the content of serotonin and its metabolite 5-hydroxyindoleacetic acid in the studied structures.

### 2.3. Na,K-ATPase Activity in the Midbrain and Cerebellum

**Figure 4:**
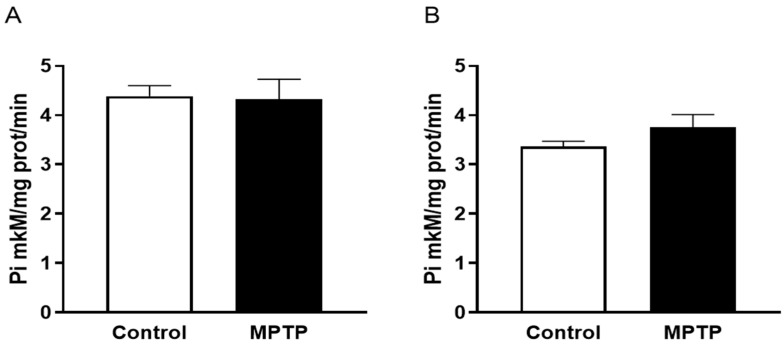
Na,K-ATPase activity in the midbrain (**A**) and in the cerebellum (**B**). N = 12 in the control group, N = 11 in the MPTP group. The data are presented as mean ± SEM. The groups were compared via an unpaired *t*-test.

Evaluating total activity of Na,K-ATPase revealed no differences in the function of this enzyme in both experimental groups in the midbrain (*p* = 0.8893, F = 2.060) (Figure 4A) and cerebellum (*p* = 0.2380, F = 6.850) (Figure 4B), which indicates the enzyme resistance to the low doses of neurotoxin and no dysregulation of membrane potential in the key structures involved in pathological processes.

### 2.4. The Evaluation of Tyrosinohydroxylase Content in the Striatum and SNpc

**Figure 5:**
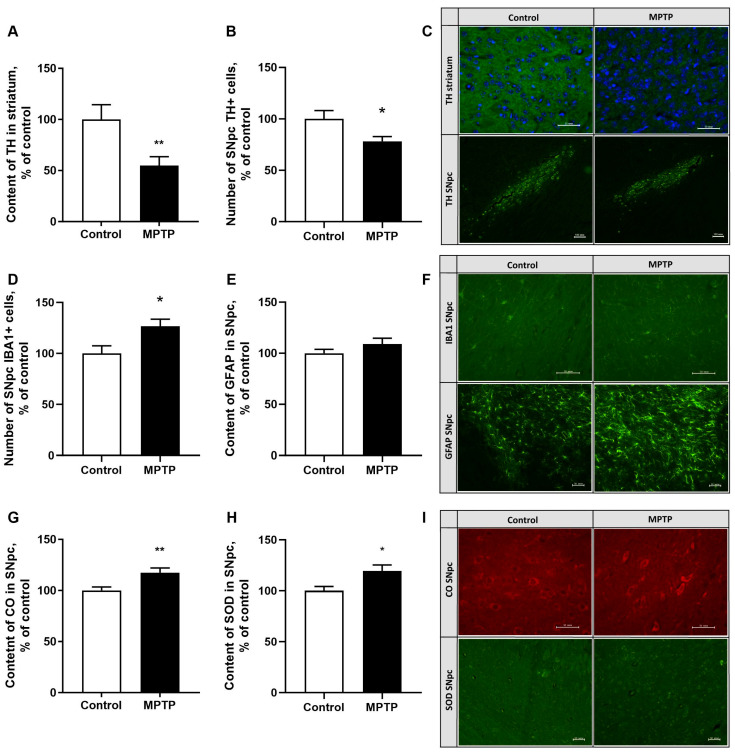
Changes in the staining intensity of tyrosinehydroxylase (TH) in the striatum as a percentage of the control in brightness gradations of an 8-bit image (**A**). Changes in the number of dopaminergic neurons in the SNpc (**B**). Changes in TH content in the striatum and in the SNpc after chronic exposure to MPTP at a dose of 3 mg/kg. The cut was made at 1.92 mm according to the Paxinos and Franklin atlas. Scale bar: TH striatum 50 µm (objective 40×), TH SNpc 100 µm (objective 10×) (**C**). Changes in the number of microglia cells in the SNpc (**D**). Changes in the staining intensity of glial fibrillary acidic protein (GFAP) in astrocytes in the SNpc as a percentage of the control in brightness gradations of an 8-bit image (**E**). Changes in the morphology of microglial cells and the content of GFAP in astrocytes in SNpc after chronic exposure to MPTP at a dose of 3 mg/kg. The cut was made at 1.80 mm according to the Paxinos and Franklin atlas. Scale bar: IBA1 SNpc 50 µm (objective 40×), GFAP SNpc 100 µm (objective 20×) (**F**). Changes in the staining intensity of cytochromeoxidase (CO) in the SNpc as a percentage of control in a 12-bit image (**G**). Change in the staining intensity of superoxidedismutase (SOD) in the SNpc as a percentage of the control in brightness gradations of the 8-bit image (**H**). Changes in the content of CO and SOD in the SNpc after the chronic action of MPTP at a dose of 3 mg/kg. The cut was made at 1.80 mm according to the Paxinos and Franklin atlas. Scale bar: CO SNpc 50 µm (objective 40×), SOD SNpc 50 µm (objective 20×) (**I**). N = 12 in the control group, N = 11 in the MPTP group. The data are presented as mean ± SEM. The groups were compared by *t*-test, *-*p* < 0.05, **-*p* < 0.01 as compared to the control group.

The damage to the nigrostriatal system was assessed by measuring the content of the key enzyme involved in dopamine synthesis—tyrosine hydroxylase—in the striatum and SNpc by immunohistochemistry. Chronic administration of MPTP caused a decrease in the numbers of TH (tyrosine hydroxylase)-positive cells by 22% in the SNpc (*p* = 0.0196, F = 2.791) (Figure 5B) and damage to axon terminals of dopaminergic neurons in the striatum by 37% (*p* < 0.01) (Figure 5A) compared to the control on day 35 of the experiment.

### 2.5. The Assessment of the Neuroinflammatory Reaction Development in the SNpc

The development of neuroinflammatory response in the SNpc was assessed using immunohistochemical staining for IBA1 (ionized calcium-binding adapter molecule 1), a microglial marker, and for GFAP (glial fibrillary acidic protein), an astrocytic marker. By day 35, chronic administration of 3 mg/kg of MPTP led to an increase in the number of microglia cells by 27% in the SNpc (*p* = 0.016, F = 1.118) (Figure 5D) as well as their activation, which manifested in decreased numbers of processes and an increased soma size compared to control. At the same time, the GFAP content in the astrocytes of the SNpc remained within the control values (*p* = 0.2009) (Figure 5E).

### 2.6. The Evaluation of Superoxide Dismutase and Cytochrome Oxidase (Isoform 1) Content in SNpc

The total content of superoxide dismutase and cytochrome oxidase (isoform 1) was assessed by immunohistochemistry. Chronic administration of MPTP for 35 days elevated the cytochrome oxidase and superoxide dismutase content in the SNpc by 17% (*p* = 0.0074, F = 1.764) (Figure 5G) and 19.5% (*p* = 0.0168) (Figure 5H), respectively. These data serve as the evidence of the activation of the antioxidant system (AS) and of the system responsible for the energy balance in the brain, indicating the existence of compensatory mechanisms that prevent OS development.

## 3. Discussion

Searching for diagnostic criteria for the early preclinical stage of PD is a task of undisputable importance in neurology. In this regard, this study aimed to evaluate the behavioral and neurochemical characteristics of the murine model mimicking pre-motor stage parkinsonism induced by a long-term 35-day administration of a low dose of the MPTP neurotoxin.

It should be noted that experimental studies employ various methods of MPTP-induced parkinsonism models, including both acute and subacute toxin administration. To date, three neurotoxin-based methods of modeling parkinsonism have been described. The acute toxic model is characterized by administering a high dose of the toxin over a single day, resulting in 60% neuronal death in the substantia nigra by necrosis. It is accompanied by a significant decrease in striatal dopamine levels, ranging from 40% to 90%, depending on the cumulative dose of MPTP, with mice exhibiting impaired gait, postural instability, bradykinesia, and a reduced motor activity [17,28]. The subacute MPTP administration protocol involves daily injections of 30 mg/kg of the toxin for 5 days, which causes apoptosis of SNpc neurons, which reaches a plateau by day 21 and is accompanied by a 40–50% decrease in striatal dopamine levels. However, this murine parkinsonism model shows a number of behavioral inconsistencies, including an increased locomotor activity in the home cage and the open field test.

The hyperactivity observed could arise from an increased activation of the dopaminergic system and increased dopamine turnover, limiting the use of this model for drug screening [17,29,30]. In our study, we employed the previously developed chronic administration model characterized by a daily administration of 4 mg/kg of MPTP over the course of 28 days [22]. At the same time, a 40–50% decrease in dopamine and TH-positive neurons of SNpc is observed in the animals’ brains, accompanied by impaired gait and the overall neurological decline, indicating the early motor stage of parkinsonism.

To detect the main signs of the premotor stage of parkinsonism, we reduced the MPTP administration dose to 3 mg/kg per day and extended the duration of administration to 35 days, in contrast to the previous study [22]. During different periods of toxin administration, we evaluated the development of Parkinson’s disease symptoms. The mice’s memory and motor control functions were evaluated within a cluster of various behavioral tests that assess spatial memory, coordination, bradykinesia, and fine motor skills. Our findings indicated an absence of motor disorders in the animals’ behavior, and only the inclined grid walking test revealed a decrease in the animal movement speed, indicating the onset of bradykinesia. We can therefore conclude that our model accurately mimics the early stage of parkinsonism.

Recently, a large number of studies have been devoted to the study of emotional and cognitive changes in animal models of PD corresponding to the premotor stage of this disease [31,32]. Depression is a common non-motor symptom in patients with Parkinson’s disease (PD), which is difficult to treat. In models of 6-OH-DA- and MPTP-induced parkinsonism, animals were found to exhibit depressive-like behavior characterized by an increase in fading time in the free swimming test, and anhedonia before the appearance of motor disorders [33,34,35]. A violation of social interaction in animals was also demonstrated against the background of 40% damage to dopaminergic neurons. The observed nonmotor manifestations correlated with a decrease in the content of dopamine in the striatum and serotonin in the hippocampus of animals [33]. It is known that when patients with PD are depressed, there is a change in serotonin levels, and a post-mortem examination revealed that patients with PD and depression had increased loss of neural cells in the serotonergic dorsal raphe nuclei compared with patients with PD without depression [36,37]. At the same time, it is known that Lewy bodies accumulate in the serotonergic cell bodies of the raphe nuclei already at the second stage of the disease, which suggests that serotonergic neurons are affected even earlier than dopaminergic neurons [36]. Based on these facts, a hypothesis of the neurochemical nature of depression has been suggested, according to which a decrease in the content of 5-HT or an increase in the inhibitory activity of 5-HT2C may be associated with a decrease in dopaminergic neurotransmission in PD patients and a subsequent worsening of mood symptoms. Low 5-HT activity in the brain of PD patients is a risk factor for depression [38]. Serotonin plays an inhibitory role in the release of DA in the striatum; therefore, a decrease in 5-HT levels may be a compensatory mechanism associated with a decrease in dopaminergic neurotransmission in PD [38,39]. The present study is consistent with the literature data provided. Thus, the introduction of MPTP for 35 days did not lead to the formation of depressive-like behavior, assessed in the tail-hanging test, and also did not lead to a change in the content of serotonin in the prefrontal cortex. Perhaps the reduction in striatal dopamine to 34% was not enough to trigger a pathological decrease in serotonin levels. However, in the model of acute MPTP-induced Parkinsonism, it has been shown that the death of SNpc neurons correlates with a decrease in serotonin levels in the hippocampus without the manifestation of a depressive-like phenotype [40].

An important aspect of the present work, which characterizes the pre-symptomatic stage of parkinsonism, was to detect spatial working memory deficits in the Y-maze test. Our results can hold the diagnostic value for the premotor and early stage of Parkinson’s disease both in experimental [41,42,43] and clinical studies [44,45], since cognitive impairment in the brain are observed along with severe motor symptoms developed. Cognitive deficits are known to be caused by the changes in cortico-striatal connections, and working memory deficit is associated with impairments in the dopaminergic system functioning [46]. Our results demonstrate a decrease in the spontaneous alternation ratio in the Y-maze test, accompanied by a significant decrease in the dopamine content in the PFC and striatum by 52% and 34%, respectively, compared to control animals that exhibited no signs of memory impairment, keeping up with other experimental studies. Despite the fact that most studies on the pathology of PD focus on the analysis of the dopaminergic system, many of the observed cognitive impairments may also be associated with the pathology of the noradrenergic system in patients with Parkinson’s disease [47]. Postmortem analysis of the brain in Parkinsonism reveals accumulation of Lewy bodies in the Locus coeruleus [48], earlier degeneration of locus coerules neurons compared to SN neurons, as well as decreased levels of frontal norepinephrine and serotonin [49,50].

By day 35 of the experiment, the characteristic features of this stage of parkinsonism were as follows: a 34% decrease in the level of striatal dopamine, accompanied by a 37% damage to the axon terminals of dopaminergic neurons in the striatum. Furthermore, there was a less pronounced neuronal death (19%) and the number of TH-positive cells in the SNpc (22%) compared to the previous data [22]. At the same time, the number of microglia cells in the SNpc were elevated by 27% indicating a neuroinflammatory response [51,52,53]. Morphological changes in microglia cells were also observed, with a decrease in the number of processes and an increased soma size. However, the study did not reveal a significant increase in GFAP content, which is commonly observed in pathological processes in the central nervous system. A slight increase in GFAP may be a predictor of astrogliosis [51,52] and arise from the compensatory mechanisms developing during the premotor stage.

The data on the increasing amount of total superoxide dismutase and cytochrome oxidase (isoform 1) indicate the activation of the antioxidant system and the system that maintains the energy balance in the brain SNpc, which implicates the compensatory mechanisms preventing OS. An increase in the superoxide dismutase activity indicates an enhanced antioxidant protection, although the previous studies have shown that the expression levels of the genes involved in ROS-fighting pathways, including superoxide dismutase, are significantly reduced in the presence of MPTP [54]. Under normal conditions, cytochrome oxidase (CO) IV-1 regulates the production of ATP synthesis according to energy requirements, but during the progression of pathological processes, the expression of CO IV-2, which produces reactive oxygen species [55], increases. Administering MPTP at a dose of 3 mg/kg leads to an increased CO IV-1 activity. These data are somewhat inconsistent with the data obtained in the model of chronic administration of 4 mg/kg of MPTP, which showed no changes in the functioning of the CO by the end of the experiment [22]. It can therefore be assumed that in this case, the increase in the first CO isoform may stem from the compensatory reactions that occur when a low dose of neurotoxin is administered.

Na,K-ATPase uses up to 40% of the ATP synthesized by the brain, which suggests possible dysfunction of this enzyme due to a decrease in ATP levels in neurodegenerative diseases [56,57]. In the MPTP-induced murine parkinsonism model, a 40% decrease in the total activity of Na,K-ATPase was observed, accompanied by a 60% decrease in the amount of striatal dopamine [19]. In addition, a significant decrease in the activity of the enzyme (up to 60%) was observed under oxidative stress conditions upon the treatment of nerve growth factor (NGF) differentiated pheochromocytoma 12 (PC12) cells with MPP+ [58]. However, the present study revealed no changes in the Na,K-ATPase function in the midbrain and cerebellum at a dopamine depletion level of 34%, which implies the secondary enzyme dysfunction accompanied by a more severe damage to the dopaminergic system arising from ATP depletion in mitochondria.

The proposed model of chronic toxin administration may have translational significance due to the slow development and long-term progression of parkinsonism. This model enables the dynamics of degeneration of the nigrostriatal pathway from axon terminals to dopaminergic neuronal bodies in the substantia nigra pars compacta (SNpc) to be accurately reproduced. This opens up new vistas for drug testing at different stages of the disease and obtaining results closely mimicking PD in humans.

## 4. Materials and Methods

### 4.1. Animals

The experiment was conducted on C57BLACK/6 mice (n = 23) aged 6–8 weeks, weighing 20–25 g at the beginning of the study. The animals were kept in standard controlled vivarium conditions (at a temperature of 22 ± 2 °C; humidity of 55 ± 5%) at Moscow State University’s Biological Faculty, with a 12-h light/dark cycle and access to food and water ad libitum. All manipulations with the animals were carried out in the daytime (from 9:00 to 20:00).

The animals were maintained according to the international standards 33215-2014 Guide for the Care and Maintenance of Laboratory Animals [59]. The work was designed and carried out in accordance with international regulations Directive 2010/63/EU of the European Parliament and of the Council of 22 September 2010 on the protection of animals used for scientific purposes, and the European Convention for the Protection of Vertebrate Animals used for Experimental and other Scientific Purposes [60].

### 4.2. Experimental Design

The early stage of parkinsonism was modeled via daily chronic administration of the MPTP neurotoxin. Animals were divided into two groups: the control group (N = 12) received 0.2 ml of saline, one injection per day, the experimental “MPTP” group (N = 11) received MPTP at a dose of 3 mg/kg of body weight subcutaneously, one injection per day, according to the schedule of 6 days on/1 off (6/1). Each animal received 35 daily doses of the neurotoxin in total.

The mice were handled for 6 days prior to the experiment to familiarize them with the testing conditions. During the experiment, the animal motor impairments were assessed once a week using the Beam walking test and Inclined grid walking tests. After 35 MPTP injections, mice locomotor activity was evaluated in the Open field test, depressive-like behavior was evaluated in the Tail-suspension test and spatial working memory was evaluated using the Y-maze spontaneous alternation test. Table 1 presents the scheme of behavioral testing.

24 h after the last MPTP injection, animals were decapitated, and their brains were removed. The prefrontal cortex (PFC: infralimbic cortex, prelimbic cortex and cingulate cortex), striatum (dorsomedial and dorsolateral striatum), midbrain, and cerebellum were isolated from one hemisphere on ice and frozen in liquid nitrogen for further biochemical analysis. Brain dissection was performed in accordance with the protocol described by Chiu et al. [61]. The other hemisphere was fixed in neutral 4% formalin for further immunohistochemical examination.

### 4.3. Behavioral Testing

The beam walking test was used to assess animals’ hypokinesia, balance, and coordination. The experiment was conducted using a 100 cm long beam resting 50 cm above the tabletop on 0.75 cm wide poles and gradually narrowing from 4 cm to 0.5 cm (OpenScience, Moscow, Russia). The animals had been pre-trained to walk along the beams for three sessions of three trials each. During experiment, the animal was placed at the starting point, and the time to traverse the beam was recorded. The animal was given three attempts, and the average walking time was calculated [62].

The inclined grid walk test was used to detect subtle motor deficits of the animals’ limbs. Testing was carried out using a 20 × 20 cm grid with a 1.5 cm distance between the rods, placed at a 45-degree angle on a horizontal surface. The upper end of the grid was attached to the home cage. Prior to the experiments, the mice had been trained to pass the test for 3 sessions of three trials each. At the beginning of the testing, the mice were placed on the lower rods of the grid, so that the animals had to climb up the grid in order to return to their housing. During the test, the total ascending time (the time from the start of the test to the moment when all animal’s paws had touched the home cage grid rods) and the number of mistakes (paws slipping between the grid rods) made by the animal were recorded. The test session was confined to 90 s. The animal was given 3 attempts, and the average test completion time was calculated [62].

The open field test was used to study the locomotor activity of mice. The experiments were carried out in a round open field with a diameter of 63 cm and a wall height of 32 cm (OpenScience, Moscow, Russia). The floor of the apparatus was divided into 3 rows of equal-area sectors, which made it possible to determine the distance traveled by the number of sectors crossed. Behavior was recorded using the RealTimer program, v. from 31.01.2009 (OpenScience, Moscow, Russia). The following parameters were evaluated over the course of 5 min: the distance traveled and the number of rearing instances.

The Tail Suspension Test (TST) was used to assess the depressive-like behavior. Each mouse was suspended by the tail 60 cm above the floor using adhesive tape placed <1 cm from the tip of the tail. The total duration of immobility was evaluated for 6 min. In this test, the “period of immobility” was defined as the period when the animals stopped resisting for ≥1 s.

The Y-maze test was used to analyze spatial working memory dysfunction. The experimental setup consists of a maze of three 30 × 5 × 12 cm arms connected at a 120° angle (OpenScience, Moscow, Russia). The animal was placed at the end of a random arm and allowed to explore the maze for 5 min freely. The number and order of the mouse entering the maze arms were recorded, and the alternation coefficient (Ks) was calculated using the formula: Ks = R/A × 100%, where “R” stands for the number of correct alternations (three consecutive entries into the maze arms without repetition), and “A” stands for the maximum possible number of alternations (the total number of entries into the arms) [34].

### 4.4. The Content of Monoamines

The content of monoamines and their metabolites was determined by high-performance liquid chromatography with electrochemical detection (HPLC-ED) in samples of the prefrontal cortex and striatum from the experimental animals. The striatum and cortex samples were homogenized in 20- and 10-fold volumes of 0.1 N of HClO_4_, respectively, added with 3,4-dihydroxybenzylamine hydrobromide at a concentration of 0.25 nmol/mL used as an internal standard. A glass/Teflon pestle homogenizer Shuett Homgen Plus (0.2 mm) (Schuet Biotec GmbH, Göttingen, Germany) was used for homogenization. The samples were centrifuged at 10,000 g for fifteen minutes at 4 °C. The content of monoamines (dopamine (DA) and serotonin (5-AT)) in the supernatant was determined using a System Gold liquid chromatograph (Beckman Coulter, Inc., Brea, CA, USA). The content of monoamines (dopamine (DA) and serotonin (5-AT)) was determined following the Yang protocol. The separation of the substances studied was carried out on a Nucleodur C18 Gravity reversed-phase column, 4.6 × 250 mm, with a pore diameter of 5 mkm (MACHEREY-NAGEL GmbH & Co. KG, Düren, Germany). Measurements were carried out using an EC3000 electrochemical detector (RECIPE Chemicals + Instruments GmbH, Munich, Germany) equipped with a ClinLab ECD cell, the Sputnik model, with a glass-carbon working electrode (+0.85 V) and an Ag/AgCl reference electrode. The MULTICHROM 1.5 software (AMPERSAND, Moscow, Russia) was used for sample registration and chromatogram processing. All reagents used for the analysis were of analytical grade. The units of measurement were expressed in nmol/g of wet tissue mass.

### 4.5. The Activity of Na,K-ATPase

Na,K-ATPase activity was determined based on the amount of inorganic phosphate released during the reaction, using a method developed by Rathbun and Betlach [63] with modifications. All operations were performed on ice. The cerebellum and midbrain samples were weighed and homogenized using a Schuett Homgen Plus glass/Teflon pestle homogenizer (SchuettBiotec GmbH, Göttingen, Germany) in a 10-fold volume of extraction buffer (0.25 M sucrose, 1 mM EDTA, 20 mM Tris, pH 7.45) containing cocktails of protease and phosphatase inhibitors (Sigma-Aldrich, St. Louis, MO, USA) added immediately before use. The homogenate was centrifuged for 5 min at 2000× *g* at 4 °C. The supernatant was transferred to a separate container and centrifuged for 15 min at 14,000× *g* at 4 °C. The synaptosomal fraction was resuspended in the isolation buffer and stored at −70 °C. To determine protein concentration, a portion of the preparation was lysed in RIPA buffer containing cocktails of protease and phosphatase inhibitors (Sigma), and protein concentration was determined using the DC Protein Assay Kit (Bio-Rad, Hercules, CA, USA). The synaptosomal fraction with a protein concentration of 1 mg/mL was incubated with 0.065% sodium deoxycholate in a cold-water bath for 30 min. The resulting Na,K-ATPase preparation (4 mkg of protein per sample) was added to the reaction medium (130 mM NaCl, 20 mM KCl, 3 mM MgCl_2_, 30 mM imidazole, pH 7.5). The reaction was initiated by adding ATP at a final concentration of 3 mM. The reaction mixture was incubated at 37 °C for 15 min. The reaction was terminated by adding 0.1 mL of chilled 3 M acetate buffer (pH 4.3) containing 3.7% formaldehyde. To determine the amount of inorganic phosphate released, 0.02 mL of 2% ammonium heptamolybdate and 0.02 mL of freshly prepared 0.3% Sn(II) chloride solution were added to the sample. The samples were thoroughly mixed and left to stand for 10 min at room temperature. The optical density of the solution was measured at 735 nm using a Synergy H1 plate reader (BioTek, Winooski, VT, USA). Na,K-ATPase activity was calculated as the difference between the optical densities of a test sample and a sample containing 4 mM ouabain.

### 4.6. Immunohistochemistry

Brain samples were fixed in formalin in phosphate saline buffer and dehydrated with ethanol and chloroform for embedding in paraffin. Series of 10 micrometer thick frontal sections were prepared using a Leica SR2000 cryotome (Wetzlar, Germany). Samples were stained using immunohistochemistry. To detect tyrosine hydroxylase (TH) (1:250, T8700 Anti-TH Rb (Sigma-Aldrich, St. Louis, MO, USA), ab112 Anti-TH Rb (Abcam, Cambridge, UK)) and microglia (IBA1) (ionized calcium-binding adapter molecule 1) (1:300, ab178847, Anti-IBA1 Rb (Abcam, Cambridge, UK)), monoclonal rabbit antibodies, cytochrome oxidase monoclonal mouse antibodies (COX) (1:200, Lot 459600-Anti-MT-CO1 Ms (Invitrogen, Waltham, MA, USA)), astrocytes (GFAP) (1:200, ab7260, Anti-GFAP Rb (Abcam, Cambridge, UK)) and superoxide dismutase (SOD) (1:200, PA1-30195, AntiSOD (Invitrogen, Waltham, MA, USA)) were used. From 12 to 18 sections were taken from each animal at a level of 0.48–1.80 mediolaterally (Paxinos et al., 2001) [64]. The preparations were analyzed and photographed using a Nikon Eclipse Ni-U microscope equipped with a Nikon DS-Qi camera. The neural cell count and intensity of immunostaining were performed using the ImageJ 1.52u program (Bethesda, MD, USA).

### 4.7. Statistical Data Processing

Data analysis was performed using the GraphPad Prism 10.0.0 (GraphPad Software, Inc., La Jolla, CA, USA). All data were checked for normality of distribution using Shapiro–Wilk test. The two-way analysis of variance (two-way ANOVA) was used to analyze the data from the narrowing beam and inclined grid walking tests followed by the Šidák multiple comparison test. The unpaired *t*-test was used to assess the differences between the two groups. The differences were considered significant at *p* < 0.05. Data are presented as mean ± standard error of the mean (MEAN ± SEM).

## 5. Conclusions

Chronic administration of the MPTP toxin at a dose of 3 mg/kg for 35 days impairs fine motor skills in animals in the absence of hypokinesia as well as their spatial memory, accompanied by dopamine depletion in the PFC. Moreover, cell death in the SNpc, decreased tyrosine hydroxylase levels, and striatal dopamine content, together with the phenotypic characteristics, indicate the onset of the early pre-symptomatic parkinsonism in experimental animals. The neurodegenerative processes observed in the SNpc are accompanied by pro-inflammatory changes in microglia, an increase in the content of CO without changes in the Na,K-ATPase function, and a compensatory increase in the superoxide dismutase expression, which indicates the activation of brain antioxidant protection mechanisms involved at the initial stages of damage.

Thus, this study allowed us to describe the premotor stage of parkinsonism, which is characterized by neurochemical changes in the dopaminergic brain structures, accompanied by pronounced cognitive deficits and the absence of motor symptoms.

## Figures and Tables

**Table 1:** The behavioral testing schedule.

Number of Animals	Day of MPTP Injection	Test
control N = 12, MPTP N = 11	0, 7, 14, 21, 28, 35	Beam walking test
control N = 12, MPTP N = 11	0, 7, 14, 21, 28, 35	Inclined grid walking test
control N = 12, MPTP N = 11	35	Open field
control N = 7, MPTP N = 6	35	Tail-suspension test
control N = 7, MPTP N = 6	35	Y-maze

## Data Availability

Dataset available on request from the authors.
